# Abortion Epizootic Cellulitis

**Published:** 1883-07

**Authors:** 


### V.—ABORTION EPIZOOTIC CELLULITIS.

REPORTED FROM DR. MC LELLAN'S CLINIC AT THE THIRD AVENUE CAR STABLES.

In this disease the eyes are greatly inflamed and the legs often swollen. When an abscess occurs, with swelling in the intermaxillary space, it is generally termed Febra Pyogenica; when the catarrhal symptoms are most prominent, Influenza. When the eyes are greatly inflamed, the legs swollen, then we have the characteristic type termed cellulitis, and these are the prominent symptoms in this case.

*Symptoms.*

Pulse extremely weak and rapid. Temperature, 104.5° F. Slight discharge from nostrils. Mucous membranes injected, submaxillary glands swollen. The œdematous swelling of the limbs is also very well marked. Slight difficulty in breathing, and on pressing in region of throat animal evinces increased pain. A diagnosis of epizootic cellulitis was therefore made.

A favorable prognosis was given, for the reason that this form, when properly treated, as a rule terminates favorably and in a comparatively short time. But frequently animals are apt to abort, as it is termed; the attack starting in all right, but suddenly coming to a terminus.

*Treatment.*

During the early stages, when the fever is a marked element in the disease, tincture of aconite in drachm doses may be given. An ounce of nitrate of potassium may be added to their drinking water. Further than this treat the symptoms. If the sore throat be a marked lesion, use stimulating liniments externally. If there is great difficulty in breathing, the head may be steamed either simple or medicated.

With great irritability of the bowels, hydrastis is often serviceable.

December 20th, 1882, at a later clinic, Dr. Hough, the surgeon in charge of the infirmary, reported that the attack aborted on the 17th inst. After the previous clinic these symptoms intensified. The catarrhal symptoms then being most prominent, with considerable discharges from the nostrils, and also from eyes. Swelling in the intermaxillary space. Mucous membranes more congested. Pulse frequent and feeble. Temperature 104° F. Legs very œdematous. Breathing, however, nearly normal.

The left lung at this time was somewhat affected; consolidation of one lobe made out positively.

Treatment at this stage of the disease calls for tonic and stimulating remedies. Aconite is now positively contra-indicated, and if administered would surely cause a fatal termination. A mixture containing Liq. Ammon. Acetatis, Tr. Gentian and Iron, in such proportions as the indications may seem to call for, are to be pushed strongly. Some advise the carbonate of ammonia in combination with camphor. Dr. McLellan finds that alcohol serves the purpose as a stimulant quite as well as anything. In the country he often uses cider brandy in gruel to stimulate the heart and aid in digestion and absorption, thus perfecting the assimulation power and adding permanent strength and tonicity to his patients.

The prognosis in this case, with active treatment should be considered favorable rather than otherwise. The swelling of the legs is in all probability due to a weak acting heart, and if that is attended to the œdema should not be regarded as in itself a bad omen. The reason of this œdema is on account of the distance from the centre and the dependency of the extremities, so that the veins and lymphatics fail to do their duty.

However, we were later informed that the animal died January 3d.

A necropsy was made, but the only positively attainable information was that one of the lungs was in a state of hepatization. Probably collateral œdema of opposite lung caused heart paralysis, and was the cause of death.

